# The Three Pillars of Natural Product Dereplication. Alkaloids from the Bulbs of *Urceolina peruviana* (C. Presl) J.F. Macbr. as a Preliminary Test Case

**DOI:** 10.3390/molecules26030637

**Published:** 2021-01-26

**Authors:** Mariacaterina Lianza, Ritchy Leroy, Carine Machado Rodrigues, Nicolas Borie, Charlotte Sayagh, Simon Remy, Stefan Kuhn, Jean-Hugues Renault, Jean-Marc Nuzillard

**Affiliations:** 1 Department of Pharmacy and Biotechnology, University of Bologna, 40126 Bologna, Italy; mariacaterina.lianz3@unibo.it; 2 Université de Reims Champagne Ardenne, CNRS, ICMR UMR 7312, 51097 Reims, France; ritchy.leroy@univ-reims.fr (R.L.); carine.machado@univ-reims.fr (C.M.R.); nicolas.borie@univ-reims.fr (N.B.); charlotte.sayagh@univ-reims.fr (C.S.); simon.remy@univ-reims.fr (S.R.); jh.renault@univ-reims.fr (J.-H.R.); 3 School of Computer Science and Informatics, De Montfort University, Leicester LE1 9BH, UK; stefan.kuhn@dmu.ac.uk

**Keywords:** natural products, dereplication, databases, spectroscopy, taxonomy, molecular structures

## Abstract

The role and importance of the identification of natural products are discussed in the perspective of the study of secondary metabolites. The rapid identification of already reported compounds, or structural dereplication, is recognized as a key element in natural product chemistry. The biological taxonomy of metabolite producing organisms, the knowledge of metabolite molecular structures, and the availability of metabolite spectroscopic signatures are considered as the three pillars of structural dereplication. The role and the construction of databases is illustrated by references to the KNApSAcK, UNPD, CSEARCH, and COCONUT databases, and by the importance of calculated taxonomic and spectroscopic data as substitutes for missing or lost original ones. Two NMR-based tools, the PNMRNP database that derives from UNPD, and KnapsackSearch, a database generator that provides taxonomically focused libraries of compounds, are proposed to the community of natural product chemists. The study of the alkaloids from *Urceolina peruviana*, a plant from the Andes used in traditional medicine for antibacterial and anticancer actions, has given the opportunity to test different approaches to dereplication, favoring the use of publicly available data sources.

## 1. Introduction

### 1.1. General Considerations

Organic natural products are produced by living organisms to ensure their own basic functional requirements through primary metabolism and to fine-tune the relationships with their surrounding through specialized or secondary metabolism. The term “natural product” (NP) generally refers to an organic specialized metabolite. NP biosynthesis is controlled by the genes and therefore depends on organism species. The biological evolution led to the preservation of some NPs across related species while others were left over. A set of species may consequently share a set of identical specialized metabolites. The taxonomic classification of species relied on phenotype comparison at the time biologists would not even dream to have access to the genome of living organisms. The identification of preserved NP structures or structural features could then assist the classification task through chemotaxonomic studies, as NP structures are part of the phenotype.

The investigation of NPs is not only bound to taxonomic studies but is motivated by the uses human beings make of them. NPs with therapeutic, organoleptic, psychoactive, poisonous, tinctorial (non-exhaustive list) properties were generally not produced to be used by humans for the purposes organisms produce them. Most of NP properties are related to their interaction with other biologically produced systems, referred to as NP targets. An NP would thus more likely interact with a target of therapeutic interest (an enzyme to be inhibited, for example) than with a randomly chosen molecule drawn from the chemical space of organic molecules, due to the co-evolution of all living species over hundreds of millions of years.

The understanding of the interaction between an NP and a target is a challenging task and is often a necessary step for the design of chemical compounds with enhanced properties [1]. This step requires a precise knowledge of the structure of the NP (and of its target) at the atomic level, a concern that converges with the one of chemotaxonomy.

Finding the structure of a compound that is already known should be, at least seemingly, much easier than the one of an unknown compound. The tentative identification of known compounds is one of the aspects of what is covered by the term “dereplication”, because earlier efforts for purification and/or structure determination have not to be replicated [2]. Undertaking dereplication in first place makes sense because an organism for which nothing is known about its chemistry may share compounds with an already studied organism with close taxonomic relationship for the reason invoked in the first paragraph. Compounds that resist dereplication may be false (known) unknowns when the employed dereplication tools fail or true (unknown) unknowns [3], for which isolation and structure elucidation tools have to be deployed [4]. The determination of the molecular structure of NPs by dereplication constitutes an important part of this article.

Dereplication is a matter of collective memory by essence. This raises the questions of what information has to be preserved and of how to do it. Proving that two substances are identical at the atomic level is currently achieved by physico-chemical methods. The data produced by the analytical instruments and the related conditions in which they are obtained, namely the meta-data, are of prime importance. By language abuse, the analytic data and their associated meta-data will be referred to here as “spectroscopic data”. If the molecular structure of compound A is known and if compound B is proved to be identical to compound A by spectroscopic data comparison, then the structure of compound B can be asserted as being also the one compound A, without having to interpret the data obtained from compound B, hence providing the expected time and effort gain. 

Obviously, structures must be preserved along with associated spectroscopic data as the end of the currently described dereplication process is the labeling of a sample with the structure of a compound (compound naming will be discussed hereafter). A theoretical dereplication strategy would be to preserve the structure and spectroscopic data of all, probably less than 400,000, known NPs to date, and to compare the data from a presumably known compound with all the preserved data. If ever possible, this approach would be highly inefficient and it would be more efficient to limit the comparison work to the compounds from organisms that are taxonomically close to the one from which the currently considered NP comes from, in the way used by NP chemists during the pre-computer age [5]. This means that a link should be preserved between a NP structure and the taxonomy of the organism(s) it originates from. It clearly appears at this point that structure description, spectroscopy and taxonomy constitutes the three pillars of dereplication. Selected aspects of each of them are detailed hereafter.

### 1.2. The Three Pilars of Dereplication

#### 1.2.1. Molecular Structures

The structure of purely organic compounds, excluding organometallic species, is remarkably well described by mathematical graphs, with atoms as nodes and bonds as edges. The idea of matching a single compound with a single structure is valid at least when a compound cannot be described by more than one tautomeric form. While InChI [6] and SMILES [7] linear notations retain all the necessary structural features of a compound, including chirality, the text-based MOL format (and the derived SDF format) is widely used as it includes atom coordinates necessary either for 2D depictions or for 3D viewing [8]. The three representation modes evoked here may coexist in order to avoid conversion operations, even though a computer tool can facilitate them [9]. Structures may be surrounded by various calculated properties (molecular formula, molecular mass, chemical classification, topological descriptors, for example) coded as tag-value pairs. The compound name may be also considered as calculated property. Aspirin is indeed acetylsalicylic acid (another compound name for the same substance) for which IUPAC [10] indicates it is 2-acetoxybenzoic acid in English but “acide 2-acétoxybenzoïque” in French and “2-(Acetyloxy)benzoesäure” in German, thus precluding any kind of simple character-by-character name comparison. Considering the name as a molecular property, a compound is better referenced by a list of synonyms rather than by a single name. A structure that is proposed to be a new one because dereplication did not prove it was already known must be searched for in the literature, a task that is simplified by looking, if possible, in comprehensive structure collections such as the one provided by CAS [11] or PubChem [12].

#### 1.2.2. Spectroscopy

Spectroscopy is considered here in the broadest sense, namely as any physico-chemical methods of characterization. This includes the methods that truly rely on the interaction between electromagnetic waves and matter (UV-visible, IR, Raman, NMR, vibrational and electronic dichroism spectroscopies, optical rotation measurements) but also mass spectrometry (MS), fusion and boiling temperature measurements, and others. Even a single optical rotation value has to be associated to meta-data, such as the nature of the used solvent, the sample concentration, the temperature, and possibly the model of the measurement device. Reporting in NMR spectroscopy is a much more complex task as it must encompass the conditions of raw data acquisition, the nature of the processing operations that lead to spectra and the feature recognition processes that produce “reduced data” (a list of chemical shift values correspond to the position of spectral peaks, for example) and ultimately contributes to molecular structure proposals [13]. The diversity of spectrometer manufacturers, each proposing its own file formats, clearly precludes the easy comparison of spectroscopic data, even though a universal, text-based format named JCAMP [14] is supported by the IUPAC but could be possibly superseded in a near future by the ADF format of the Allotrope foundation [15]. The result of spectroscopic analysis is only meaningful if the link between data and compound structure is preserved, possibly leading to spectra interpretation, thus making possible to associate a particular spectral feature (the mass of a molecular fragment in MS) and a structural feature (a fragment of a molecular structure). There is no easy way to access the spectroscopic data of known natural products [16]. Most of visible efforts in this direction were devoted to the characterization of primary metabolites in the perspective of metabolomic studies [17,18].

A set of NMR and of MS*^n^* spectra constitute a better way to identify a known compound than a fusion temperature and an optical rotation value, even though the two latter may suffice to rule out an incorrect structure hypothesis. Dereplication by MS-based methods has earned a high level of interest with the advent of MS^2^-driven molecular network analysis [19,20]. Alternatively, a molecular (elemental) formula deduced from high-resolution MS (HRMS) associated to 1D and 2D NMR spectra may suffice to identify a known compound with a high level of confidence if reference data are available. A workaround to the lack of experimental spectroscopic data can be found, more or less accurately depending on the analytical technique, by means of computerized prediction tools. Dereplication of NPs based on ^13^C-NMR predicted data has been reported and discussed [21,22]. Such predictions may be carried out by various software, including proprietary or free methods, available on local computers or through web interfaces, with possible automated use or not, and with performances that can be difficult to evaluate. CNMR Predictor, NMRPREDICT, ChemDraw, nmrshiftdb2 are such software, among which nmrshiftdb2 may be used for free in an automated may on a local computer while CNMR Predictor is a commercial product renowned for its accuracy.

#### 1.2.3. Taxonomy

Taxonomy of living beings is a science in permanent evolution, where the findings of molecular biology separate species that were assumed to be close parents according to phenotype similarity and possibly finds similarities where none was apparent, while taxa names might evolve during time. Tools such as “NCBI Taxonomy Browser” [23] and “Tree of Life” [24] are of great help to navigate through taxonomic information and to locate species belonging to a given taxon. Answering the question of which species produce a given compound and of which compounds are known to be produced by an organism of a given species is possible by means of the Dictionary of Natural Products (DNP) with limitations inherent to a commercial product.

#### 1.2.4. Databases

The results of the chemical study of living beings are diluted among a profusion of specialized scientific journals. The construction of a collective memory about NPs is not a spontaneous process, so that the initial dilution of results has to be counterbalanced by efforts for the re-concentration of the knowledge at some well-defined places named databases. Databases that link structural, spectroscopic and taxonomic knowledge should constitute the basis of well-managed NP chemistry. A must-read article recently published focuses on where to find data about NPs in 2020 [25]. Open databases for NP research containing structural and spectroscopic data were reported earlier in [26]. The grouping of structural, taxonomic, and experimental spectroscopic data of natural products was undertaken in the ‘90s in the framework of the SISTEMAT project [27]. The data and software resulting from this visionary undertaking are unfortunately not accessible to the general public [28]. Other databases dedicated to the study of NP chemistry always miss some aspect. Biological activity studies are purposely left aside in this article as they do not constitute an entry point for dereplication. As well, bibliographic databases are not considered here, as the creation of NP databases from primary literature is not discussed, even though everyone understands that this is an important aspect of NP research.

## 2. Results

This article reports the availability of a computer software for the creation of taxonomy-focused NP databases named KnapsackSearch and of a database named PNMRNP, exemplified by the study of alkaloids from *Urceolina peruviana*.

### 2.1. KnapsakSearch

The KNApSAcK website exposes multiple databases searchable by organism name, metabolite name, and other commonly used compound identifiers [29]. Searching KNApSAcK for a given genus name displays a series of lines, each one showing a compound identifier, the related CAS identifier, metabolite name, molecular formula, molecular weight, and the name of the species in which it was reported. A genus name refers to a set of species names and each species is related to a set of compounds, each one, as discussed earlier, being possibly present in organisms from different species. Directly querying KNApSAcK for family names of organisms fails. About 54,000 compounds are referenced in KNApSAcK, thus giving access to an incomplete but still non-negligible part of the chemical space of NPs.

When starting the chemical study of an organism, the search for taxonomy-related ones may not be limited to those of the same species and may extend to the entire family, to parts of it, or to super-sets of it. The goal of the KnapsackSearch (KS) project is to process a list of user-defined genera to produce a list of chemical compounds that are related to one or more of these genera. The result is obtained as an SDF file, so that each compound is associated to a 2D molecular structure with chiral center flags and to a taxonomic and a spectroscopic description. The SDF chemistry file format is not a database format by itself but is sufficiently widespread to be read by most of chemistry software and computational toolkits. The source code of KS is made of Python scripts that rely on the RDKit library of functions for cheminformatics [30]. The freely available EdiSDF software is useful for the viewing of 2D structures and related tag-value pairs [31]. 

The workflow of KS (Figure S1) starts with the collection of all pairs made of a compound identifier and a binomial name obtained as replies to queries for the queried genus names. Each compound identifier (C_ID) is associated to a list of organism binomial names. As an example, Figure S2a shows the beginning of the list of compounds (columns 1–5) and organisms (column 6) related to genus *Galanthus*. C_IDs are then used as keys for compound search. Figure S2b shows the result of such a query for galanthamine, C_ID C00001570. The resulting in data aggregates containing a compound name, a molecular formula, a molecular weight, a CAS number (if any), an InChI string, the InChIKey hashed form of the InChI, and a SMILES string. The latter is decoded to produce atom and bond lists reshaped as a 2D MOL block. A compound is validated at this stage if the InChI calculated from the MOL block is identical to the one given by KNApSAcK. Molecular formula calculated from the MOL blocks are also compared to the KNApSAcK ones because the latter always lack the electric charge indication if there is one and because they may correspond to [M+H]^+^ ion formula; in these cases, the retained molecular formula are deduced from the ones of [M+H]. All InChiKeys are recalculated as it may happen that compounds with different C_IDs yield identical InChIKeys. Such compounds are withdrawn from the regular compound list and processed separately to produce compound aggregates in which the alternative attributes, such as C_IDs and names, are joined together. Each compound is then associated to the taxonomic information retrieved during the first stage of the data collection process and to the ^13^C-NMR chemical shifts as predicted by the nmrshiftdb2 software. The data record of galanthamine, as displayed by the EdiSDF software, is presented in Figure S2c.

The source code of KS is freely available [32] and a few KS-generated SDF files are given with the corresponding lists of organism genera related to a taxonomic family. The ^13^C-NMR chemical shifts included in KS files may be reformatted to be imported by NMR spectroscopy software by ACD/Labs and to facilitate compound selection according to chemical shift values, thus allowing the user to benefit from the easy prediction of ^13^C-NMR chemical values on a massive scale (massive meaning without one-by-one manual operation on structure records) by nmrshiftdb2 and from the friendly graphical interface of software from ACD/Labs. The future of the web-based approach to family-focused NP databases in KS obviously relies on the continuation of the KNApSAcK web service [33]. Database, service, and software discontinuations obviously constitute serious threats, whatever the considered domain of scientific activity.

### 2.2. Predicted NMR Data for Natural Products (PNMRNP)

This section reports the transformation of a discontinued NP database, the Universal Natural Product Database (UNPD), into a “two-pillar” NP database, PNMRNP, in which biological taxonomy data is missing. Chemical classification is tentatively proposed as a remedy to this lack. The initial data used in this process is a set of Comma Separated Value (CSV) files from UNPD, provided as a part of the In Silico Data Base (ISDB) dedicated to MS-based dereplication [34]. Most of the data transformations were carried out using RDKit and locally developed Python scripts.

The Comma Separated Values (CSV) files from UNPD contain SMILES and InChI character strings as structure descriptors of NPs (213,210 compounds). Attempts to decode the SMILES chains led to the detection of a non-negligible amount of badly formed chains, so that only InChIs were considered for 2D structure generation with retained chirality information. A set of 43 compounds was discarded, containing duplicate or organometallic or inorganic compounds.

Decoding an InChI is achieved through the dedicated software library linked to RDKit and may result into unexpected results. For example, aliphatic amides were reconstructed from their InChI as their iminol tautomer, which is correct because all tautomers of a given molecule share the same InChI. Transforming aliphatic iminols into alphatic amides was undertaken using a chemical transformation rule coded as a reaction “SMILES arbitrary target specification” also known as reaction SMARTS or SMIRKS [35]. A set of such rules was applied to fix unlikely tautomeric forms. This step would have benefited from the application of a recent molecule standardization software related to RDKit [36].

RDKit does not handle the axial chirality of substituted allenes or spirans, possibly resulting in incorrect structures upon InChI decoding. Structures of compounds for which the InChI to structure conversion and back-conversion to InChI (the so-called “round-trip”) fails to be consistent were tentatively obtained by means of the ChemDraw software driven by a python for win32 script. An identifier resolution using the Chemical Identifier Resolver (CIR) from the US National Health Institute (NIH) is attempted in case of persisting failure [37]. After final checking of round-trip consistency, the nmrshiftdb2-predicted ^13^C-NMR chemical shift lists [38] were appended to compound data, resulting in a SDF file containing 211,280 records.

Even though the initial CSV files assigned a chemical name to some of the compounds in UNPD, an alternative naming procedure was carried out. The PubChem website offers a file that relates InChI and PubChem Compound Identifier (CID) and another one that relates CID and synonym lists. A set of synonyms was associated by this means to the PNMRNP compounds that are named in PubChem.

The assignment of chemical classification data to NPs in PNMRNP does not replace genuine but unavailable biological taxonomic data but may assist NP chemists to reduce the size of the chemical space to investigate when facing a dereplication problem. The link between biological and chemical taxonomy was already exploited in SISTEMAT [39]. The production of chemical classification data constitutes a remedy to the absence of a way to associate easily and at no cost a set of living organism names to an NP identifier. Chemical classification in itself is a fuzzy concept. Discussing about the definition of an alkaloid may result in an answer such as “an alkaloid is like my wife. I can recognize her when I see her, but I can’t define her” [40]. Two independent classification systems are available in PNMRNP, one (CL1) is the result of a locally developed attempt that is not comprehensive but that may meet some needs while the other one (CL2) relies on the well-established ClassyFire software [41].

Chemical classes in CL1 are defined according to the presence of specific substructures (subgraphs or molecular graphs) and are identified using SMILES that are interpreted as SMARTS [42]. Chemical classification is organized in PNMRNP with four levels, so that menthol is reported to be a secondary metabolite, a terpene, a monoterpene, and a menthane compound. More precisely substructures are identified as deriving from primary metabolites (identifier: 01) such as amino-acids, sugars, or lipids and are otherwise classified as being specifically related to secondary metabolites (identifier: 02). Terpenes (02-02) include monoterpenes (02-02-01) that share the menthane skeleton (02-02-01-001). Sugar containing compounds (01-01) were identified through a set of 1296 SMILES chains covering open chain and cyclic sugars with possible features such as deoxy- and amino- substitution (63 classes of sugars, overall). Hexopyranoses (01-01-14), with their five asymmetric carbons, thus featuring alpha- and beta-anomeric forms, are identified by a set of 32 SMILES chains to which a generic one without chirality indicator is added. The rather ubiquitous α- and β-d-glucopyranose molecular sub-units are identified as 01-01-14-005 and -006, respectively. The idea of searching for sugars in NPs was put into practice recently in the framework of the COCONUT NP database development and the possible *in silico* deglycosylation [43]. The CL1 data items in PNMRNP include the lists of atoms concerned by each detected substructure. A part of the classification was inspired by “Pharmacognosy”, a book by J. Bruneton [44], and another part from the skeleton library included in the resource files of the LSD software, a library itself borrowed from the SISTEMAT knowledge base [45]. The catalog of SMILES that resulted from the CL1 effort toward a chemical classification of NPs is available as a supplementary information file in Excel format.

Chemical taxonomy in the second classification system (CL2) in PNMRNP results from replies to queries sent to the web interface of ClassyFire. This system deals with chemistry as a whole, distinguishing between organic and inorganic compounds at the first level, named “Kingdom” by reference to the classification of living beings. The overall hierarchy of chemical classes covers up to eleven levels. The recently reported classification tool named NPClassifier specifically targets NPs [46]. Classification CL2 was introduced with version 2 of PNMRNP [47]. The link between biological and chemical classifications is highlighted by considering that a molecule can be recognized by ClassyFire as a *Strychnos* alkaloid (*i.e.*, from a plant of the *Strychnos* genus) on a sole structural basis, without any reference to its source, possibly natural or synthetic. The natural origin of so-called “organic” compounds has become difficult to ascertain without resorting to proprietary databases, so that a NP-likeness score, a calculated molecular property, is invoked in order to evaluate to which extent a natural product is natural [48]. This approach fits with the current belief according to which a human being is better known by the algorithm of a popular social network than by her- or himself.

### 2.3. CSEARCH

The web interface of CSEARCH was also considered for NP structure dereplication besides of KS and PNMRNP. The CSEARCH web server accepts requests made of a list of ^13^C-NMR chemical shifts, at best with each value associated to a multiplicity indication (number of attached hydrogen atoms, as deduced from DEPT or multiplicity-edited 2D HSQC spectra) and returns within a few minutes a list of structures sorted in the decreasing order of likelihood, proposed from a database containing several tens of millions of compounds and their predicted chemical shift values [49]. This database mostly contains structures of synthesized molecules and has no built-in concept of NP, resulting in hard to exploit results if the query is not accurate enough but may also give the solution of the submitted problem ranked in the first places, if not in first place.

### 2.4. Databases and Dereplication

To sum up briefly, KnapsachSearch may be considered as a part of a “two-pillars and half” approach to dereplication, while a “true three pillars” would have been achieved if spectroscopic data were of experimental origin instead of being predicted. PNMRNP can be qualified as “two-pillars” with its predicted spectroscopic data (a half-pillar) and biological taxonomy replaced by chemical taxonomy (a second half-pillar). The “one-pillar and half” NMRPREDICT/CSEARCH approach, dealing with structures and predicted ^13^C-NMR spectroscopy only, should be considered before any other one, if pertinent. A tentatively exhaustive (and even more than that) source of NP data, COCONUT, collects structures from various sources to propose a publicly available document-oriented database of about 400,000 compounds, some of them being clearly not so natural. COCONUT version 1 was a “one-pillar” database, devoid of spectroscopic and taxonomic data but was recently supplemented with chemical classification (a half-pillar) and could be possibly supplemented in the future with predicted spectroscopic data (another half-pillar) to provide a useful “two-pillar” tool for NP structural dereplication.

### 2.5. Application to the Alkaloids of Urceolina peruviana (Amaryllidaceae)

**Figure 1:**
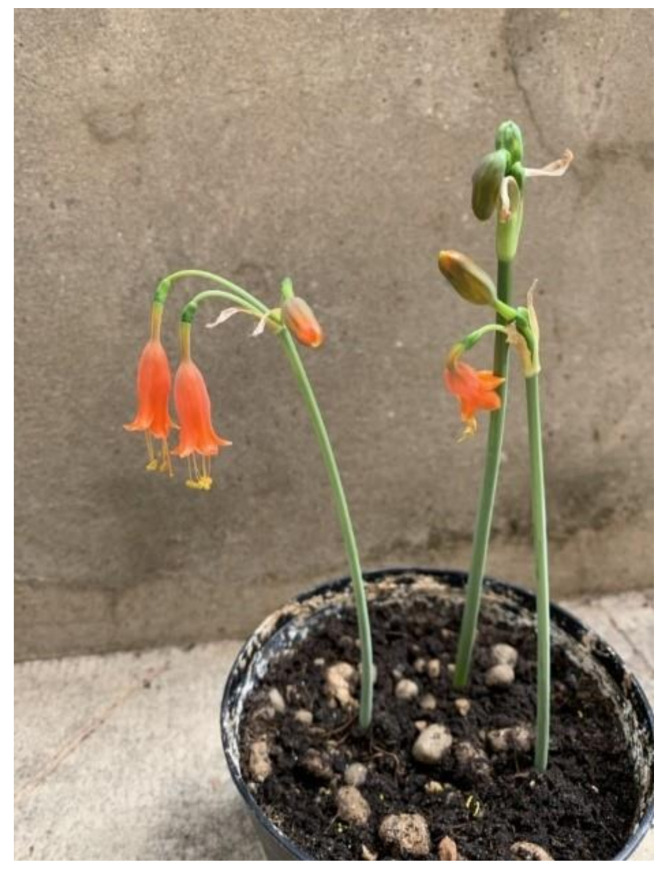
*Urceolina peruviana*.

*Urceolina peruviana* (C. Presl) J.F. Macbr., also known as *Stenomesson miniatum* (Herb.) Ravenna, is a bulbous perennial plant, which grows wild in the Andean regions of Peru and Bolivia (Figure 1). It has a scape up to 40 cm long, an umbrella of six or more red or orange tubular flowers, blooms in the spring or summer, the leaves are narrow, long until 28 cm. 

There is scarce information on this species of Amaryllidaceae in the scientific literature. The only article about the alkaloid composition of its bulbs was written in 1957 by Boit and Döpke, who reported the identification of three alkaloids (tazettine, haemanthamine and lycorine) and two others that could be traced back to nerinine and albomaculine [50]. Girault, in his book “Kallawaya, guérisseurs itinérants des Andes: recherches sur les pratiques médicinales et magiques”, on a survey carried out in the Andes on the uses of medicinal plants by the indigenous South Americans, mentions *Urceolina peruviana* whose fresh bulbs were mixed with pork or llama fat and used in the form of ointment to treat tumors and abscesses [51]. Amaryllidaceae alkaloids constitute a set of about 600 compounds, some of them, such as galanthamine, having been intensively studied for their therapeutic action [52]. The present article illustrates the use of the aforementioned NMR-based dereplication tools by the study of *U. peruviana* and on its alkaloids.

The freeze-dried bulbs of *U. peruviana* were ground before being subjected to extraction. Extract 1 (11 mg) resulted from a non-selective solid-liquid extraction of a single bulb by methanol followed by acid-base liquid-liquid extractions for basic compound isolation. Extract 2 (20 mg) was obtained by lixiviation of alkalinized powder from a single bulb by EtOAc followed by acid-base liquid-liquid extractions according to patent [53]. The method used for the preparation of extract 2 was also applied on 270 g of dry bulb powder to yield 2.742 g of extract 3. A comparison of 1D ^1^H- and ^13^C-NMR spectra of extracts 1, 2, and 3 is provided in Figure S3.

Crude extract 3 was fractionated by Centrifugal Partition Chromatography (CPC) in the so-called “pH-zone refining” development mode, which is particularly adapted to the preparative scale fractionation and purification of H^+^ ion exchanging compounds, without resorting to a solid-state chromatographic support. Emergence order of analytes from the CPC column depends on their acidity constant (K_a_) and on the distribution constant (K_D_) of their neutral form between the two liquid chromatographic phases. The chromatogram looks like trapezoidal blocks of analytes separated by steep boundaries, the so-called shock layers and forms an isotachic train of analytes [54]. The fractionation process led to 13 fractions, hereafter named A1 to A13, among which A4, A7, A9, and A11 were each found to contain a highly major compound. Purity and content of fractions A3 and A5 were very similar to the one of A4. Fraction A1 had a very low mass and a high complexity and was therefore not studied further. Fractions A2, A6, A8, A10, A12 and A13 are “intermediates” and concentrate minor compounds between the shock layers of the trapezoidal zones corresponding to the emergence of the major compounds of the injected sample. 

**Figure 2:**
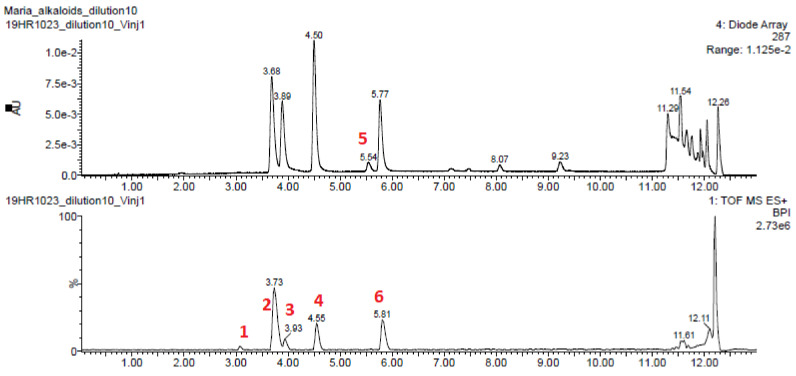
LC-HRMS ESI^+^ analysis of extract 2, UV detection (top) and ion current intensity (bottom). HRMS data are compatible with [M+H]^+^ ions of formula [C_19_H_26_NO_5_]^+^ (peak 1), [C_18_H_22_NO_5_]^+^ (peak 2), [C_16_H_18_NO_3_]^+^ (peak 3), [C_17_H_20_NO_4_]^+^ (peak 4), [C_18_H_22_NO_4_]^+^ (peak 5), [C_19_H_24_NO_5_]^+^ (peak 6).

**Figure 3:**
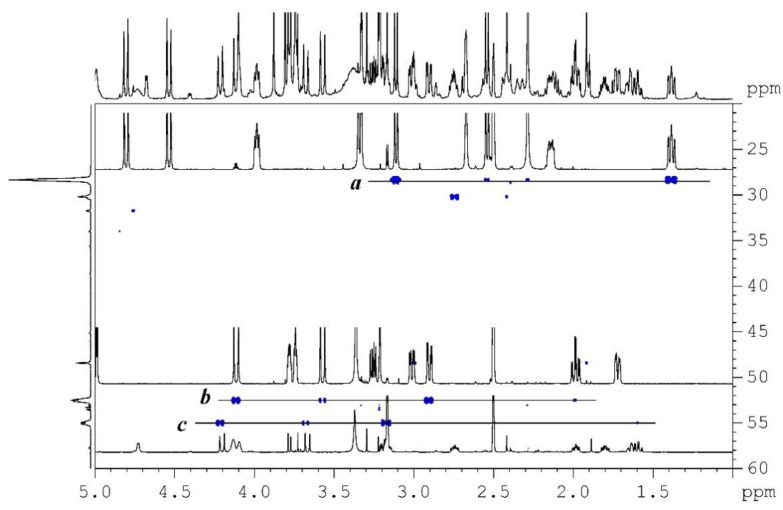
The ^1^H-^15^N HMBC spectrum of extract 3. The projection on the ^15^N chemical shift axis provides of rough estimation of extract complexity. Traces *a*, *b*, and *c* are the ^1^H-NMR spectra of tazettine, haemanthamine, and crinine recorded from fractions A4, A9, and A11, respectively.

The LC-HRMS analysis of a crude alkaloid extract 2 of *U. peruviana* monitored by UV absorbance at 287 nm showed 4 major peaks, to which molecular formula were assigned through accurate mass analysis of the [M+H]^+^ ion: C_16_H_17_NO_3_ (peak 3), C_17_H_19_NO_4_ (peak 4), C_18_H_21_NO_5_ (peak 2),and C_19_H_23_NO_5_ (peak 6) as indicated in Figure 2. Two other minor peaks, one visible in the ion-current chromatogram and the other one in the UV chromatogram were also considered for further analysis, associated to molecular formula C_19_H_25_NO_5_ (peak 1) and C_18_H_21_NO_4_ (peak 5), respectively. The LC-HRMS analysis of crude extract 3 results in the same list of formulas but with C_18_H_21_NO_4_ replaced with C_18_H_19_NO_4_ and with C_18_H_18_N_2_O_4_ and C_19_H_26_N_2_O_5_ as supplementary proposals; the two latter suggest the presence of compounds containing two nitrogen atoms, a feature that is not common among Amaryllidaceae alkaloids and the pertinence of which was not ascertained. The ^1^H, ^13^C, ^1^H-^1^H COSY, ^1^H-^1^H ROESY, ^1^H-^13^C multiplicity-edited HSQC, and ^1^H-^13^C HMBC NMR spectra of most of fractions from extract 3 were recorded. A ^1^H-^15^N HMBC spectrum of extract 3 was also recorded, also offering a rapid and rough estimate of extract complexity by inspection of the projection of this 2D spectrum on the ^15^N chemical shift axis (Figure 3).

Database creation was undertaken prior to and during the course of *U. peruviana* compound identification. The search by means of KS for the compounds reported in KNApSAcK and related to 67 genera from the Amaryllidaceae family resulted in 249 structures, among which 209 contained at least one nitrogen atom and were thus considered as possible alkaloids. These structures were imported by ACD/Labs “C+H NMR Predictors and Database” software as a new database and semi-automatically supplemented with ACD/Labs-predicted ^1^H- and ^13^C-NMR data by means of the protocol reported in Figure S4 to produce database DB1. The same set of 209 records, each including nmrshiftdb2-predicted ^13^C-NMR data, was imported by the same ACD/Labs software after appropriate reformatting of the writing of chemical shift values to yield database DB2. Six small databases containing 2 to 15 records where derived from DB2 by selecting the molecules according to the molecular formula obtained by LC-HRMS analysis of extract 3, after having verified that no compound in DB2 contains two nitrogen atoms. Database DB3 was created by the same process as DB2 but starting from the 211,280 records of PNMRNP. The latter has also been filtered to retain compounds that include one of the eight substructures that are commonly found in Amaryllidaceae alkaloids [55] (Figure S5) to give DB3′, with 635 structures. A collection of 693 compounds was created from COCONUTv1 and named DB4, retaining the compounds that contain one of the eight Amaryllidaceae substructures after an initial step that selected 109,638 compounds with more than 12 carbon atoms and with one or two nitrogen atoms. DB4 was supplemented with ^13^C-NMR chemical shifts from nmrshiftdb2 and formatted as an ACD/Labs database.

#### 2.5.1. Fraction A4, Major Compound

**Figure 4:**
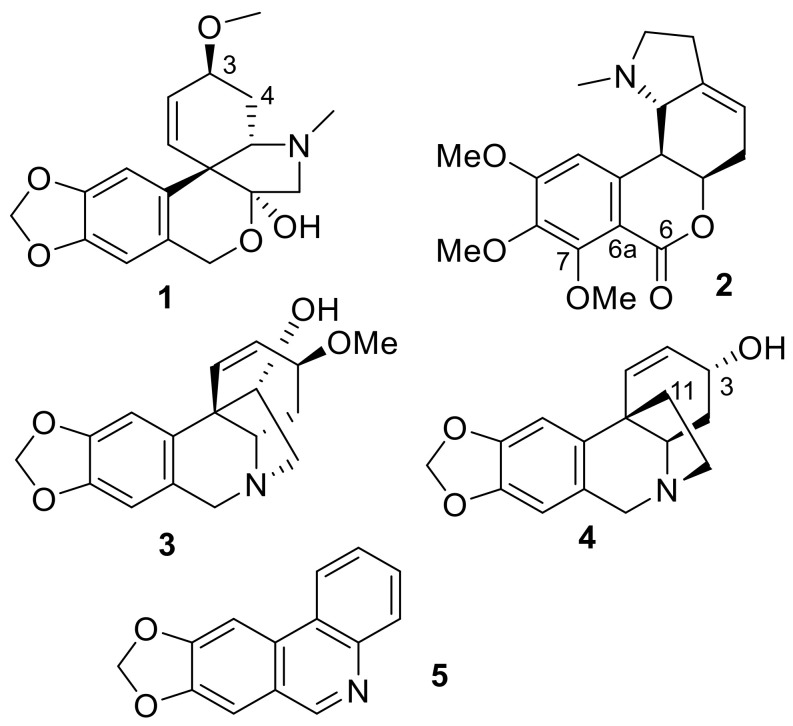
Structure of tazettine **1**, albomaculine **2**, haemanthamine **3**, crinine **4**, and trisphaeridine **5**.

This compound is also the major compound in fractions A3 and A5. The CSEARCH algorithm succeeded to retrieve tazettine **1** (structure in Figure 4) as a likely compound from the list of the 18 ^13^C-NMR chemical shifts and multiplicities from fraction A4. The molecular formula was constrained to include only C, H, N, and O atoms with a molecular mass comprised between 250 u and 400 u. Only a single chemical shift value was considered slightly unsatisfactory with δ_C_ 29.6 predicted by CSEARCH at position 4 and δ_C_ 25.9 observed (full atom numbering is reported in Figure S5). The analysis of the NMR spectra led to the identification of an aromatic ring substituted by a methylenedioxo bridge, a N-Me group, an ether O-Me group, and a hemiacetal group. The list of the C_18_H_21_NO_5_ Amaryllidaceae alkaloids in the KNApSAcK database contained two compounds among 12 that shared these NMR-derived structural features. The HMBC correlations of the ^1^H-NMR signal of the OH group lead to retain only the planar structure proposed for compound A4. None of the five C_18_H_21_NO_4_ Amaryllidaceae compound structures present in the KNApSAcK database satisfied the NMR-derived constraints. The proposed planar structure is the one of tazettine and criwelline, which are epimers at position 3 [56]. CSEARCH ranked the 6-OMe criwelline in second position. Tazettine was retained as the structure of the major compound in fraction A4 after the analysis of the ROESY spectrum and the measurement and ^1^H-^1^H coupling constants. Its molecular formula relates it to peak 2 in the chromatograms in Figure 2.

#### 2.5.2. Fraction A7, Major Compound

The CSEARCH algorithm failed to retrieve a likely structure from the list of the 19 chemical shifts drawn from the ^13^C-NMR spectrum of fraction A7. The molecular formula was constrained to include only C, H, N, and O atoms with a molecular mass comprised between 300 u and 400 u. Only two C_19_H_23_NO_5_ molecular structures of compounds from Amaryllidaceae were found in the KNApSAcK (DB2) database, among which only one contained three methoxy groups bound to an aromatic ring. This structural constraint was derived from the presence of three methyl signals in the ^1^H-NMR spectrum that correlate in the HMBC spectrum with signals of aromatic carbons. This planar structure was confirmed by all available NMR data. None of the two C_19_H_25_NO_5_ Amaryllidaceae compound structures present in the KNApSAcK database satisfied the NMR-derived constraints. The retained structure was indeed present in the solutions proposed by CSEARCH, but with a poor ranking, due to the low-quality matching between the experimental (δ_C_ 161.4, 110.9, and 155.0) and the predicted (δ_C_ 166.9, 103.1, and 156.6) chemical shifts for carbons at positions 6, 6a, and 7, respectively. Prediction by nmrshiftdb2 gave values of δ_C_ 169.8, 108.9, and 161.2 while CNMR Predictor (ACD Labs) gives δ_C_ 162.1, 111.3, and 157.1 at the same positions. The proposed structure is the one of albomaculine **2** (structure in Figure 4). Its molecular formula relates it to peak 2 in the chromatograms in Figure 2.

#### 2.5.3. Fraction A9, Major Compound

The list of 17 ^13^C-NMR chemical shifts and associated multiplicities was submitted to a spectral similarity search through the CSEARCH web interface. The molecular formula of candidate structures was constrained to include only C, H, N, and O atoms, accounting for a molecular mass comprised between 250 u and 350 u. A structure without chirality information was given as best solution, with a mean deviation of δ_C_ 1 between experimental and CSEARCH-proposed chemical shift values. KNApSAcK was also considered for the identification of the major compound in fraction A9 as a possible alternative to CSEARCH. KNApSAcK (DB2) contains 11 molecules from Amaryllidaceae with molecular formula C_17_H_19_NO_4_, the only one found by LC-MS of the total alkaloid extract accounting for 17 ^13^C resonances. From NMR data, compound A9 contains an aromatic ring with a methylenedioxo substituent and hydrogens in para position, a carbon-carbon double bond between two CH groups, and a methoxy group attached to an aliphatic carbon. The only two compounds that fit with these constraints are crinamine and haemanthamine, who present the same planar formula as the one proposed by CSEARCH. This planar structure was confirmed by the analysis of all available NMR data. The analysis of the ROESY spectrum and the ^1^H-^1^H coupling constants led to the identification of haemanthamine **3** (structure in Figure 4). Its molecular formula relates it to peak 4 in the chromatograms in Figure 2.

#### 2.5.4. Fraction A11, Major Compound

The ^13^C-NMR spectrum of fraction A11 shows 16 peaks from a major compound whose positions were used as search keys in the CSEARCH data base. The molecular formula was constrained to include only C, H, N, and O atoms with a molecular mass comprised between 250 u and 400 u. The most likely proposed structure was the one of crinine **4**, C_16_H_17_NO_3_ (structure in Figure 4). Only a single chemical shift value was considered slightly unsatisfactory (δ_C_ 40.0 predicted by CSEARCH, δ_C_ 44.2 experimental, at position 11). The KNApSAcK database of Amaryllidaceae compounds contains four compounds for this molecular formula, and only three that contain four aromatic or olefinic methine groups: crinine, vittatine, and epivittatine which only differ by the absolute configuration of asymmetric centers. More precisely, crinine and vittatine are two enantiomers, for which unambiguous identification would rely on chiroptical methods. The same situation holds for epi-crinine and epi-vittatine, epimers of the former at position 3. The identification the correct epimer was obtained by the detailed analysis of J-coupling values supported by the 2D ROESY spectrum. A comparison of the ^13^C-NMR chemical shift values in A11 with those published for synthetic crinine and epi-crinine supports our conclusion [57]. NP identification up to the absolute configuration by optical rotation measurement is possible for pure or highly major compounds but is not possible for minor compounds in fractions without isolation. Its molecular formula relates it to peak 3 in the chromatograms in Figure 2.

#### 2.5.5. Fraction A2, a Minor Compound

Fraction A2 contains a major compound, tazettine **1**, which is also the very major compound in fractions A3–A5, and many minor compounds. The ^1^H-NMR spectrum of fraction A2 shows an isolated singlet at δ_H_ 9.16 that was used as an entry point for compound identification. This highly deshielded proton is directly bound to a methine carbon at δ_C_ 151.83 according to HSQC data and is surrounded by carbons at δ_C_ 100.36 (CH), 105.40 (CH), 122.82 (C), 124.03(C), 129.6 (C), and 143.74 (C) according to HMBC data. Querying for δ_H_ 9.16 ± 0.2 in DB1 (the only one among our DBs with predicted ^1^H-NMR data) resulted in three candidate structures: angustine, vittacarboline, and trispheridine (or trisphaeridine). Searching then for δ_C_ 100.36, 105.4, 122.82, 124.03, 129.6, 143.74, and 151.83 with a 5 ppm tolerance resulted reduced the list of candidates to trispheridine **5** only (structure in Figure 4). Using DB2 and DB3 avoided to rely on proprietary NMR chemical shift prediction. Querying DB2 for the same list of seven ^13^C-NMR chemical shift with a 2 ppm tolerance yielded deoxylycobetaine chloride, trispheridine, and vasconine as proposals. A reduced tolerance of 1 ppm resulted in trispheridine only, thus also proving the good quality of the prediction by nmrshiftdb2 for this compound. Querying DB3 for the same seven chemical shift values with a tolerance of 2 ppm resulted in 628 compounds among which 124 contain at least 12 carbon atoms and 1 or 2 nitrogen atoms, using C(12-100) H(1-100) N(1-2) O(1-100) as molecular formula filter. Trispheridine is present in this compound list but reducing the number of hits would require supplementary constraints, thus demonstrating the usefulness of taxonomy-based filtering for dereplication. The presence of trispheridine in fraction A2 and its NMR spectra assignment was confirmed by further studies. Searching in DB3′ or in DB4 for trispheridine cannot be successful because its structure does not fit with any of those used in the definition of what an Amaryllidaceae alkaloid should be, even though this compound is present in the PNMRNP and COCONUTv1 database. The ClassyFire algorithm itself does not consider trisphaeridine as an alkaloid but NPClassifier identifies it as an Amarylidaceae (*sic*) alkaloid. Its NP-likeness is −0.08, a value that would make it slightly closer to a non-NP (lowest value is −5) than to an NP (highest value is +5). Exploring the philosophical implications of this observation is left as an exercise to the reader.

#### 2.5.6. Database Searches

The structural identification of compounds A4, A7, A9 and A11 reported hereabove was carried out using lists of ^13^C-NMR chemical shifts that were unambiguously drawn from spectra due to high sample purity (Table S1). After this study, a question arose about the possible results of an identification process solely relying on these lists, without any other NMR information source, only taking into account the possible molecular formula derived from LC-MS data acquired on crude extract 3. The chemical shift lists were used as search keys in DB1 (209 structures from KNApSAcK), DB2 (209 structures from KNApSAcK), DB3 (211,280 structures from full PNMRNP), DB3′ (635 structures from PNMRNP filtered for Amaryllidaceae-type alkaloids), and DB4 (693 structures from COCONUTv1 filtered for Amaryllidaceae-type alkaloids) with predicted chemical shifts by ACD/Labs software in DB1 and predicted by nmrshiftdb2 in all other DBs. All DBs were formatted for being read by the ACD/Labs DB software so that the same search tool can be used for compound identification. The poor prediction of a single chemical shift in the targetted compound may result in a global failure of the search, to which it can be remedied either by decreasing the number of experimental chemical shifts to be taken into account or by increasing the allowed chemical shift deviation. Table S2 shows the influence of these parameters on the number and nature of solutions, it illustrates the difficulty of identifying pure compounds without ambiguity solely on the basis of lists of ^13^C-NMR chemical shift values and molecular formula.

## 3. Materials

### 3.1. Chemicals

Acetonitrile (CH_3_CN), methanol (MeOH), methyl-tert-butyl ether (MtBE), chloroform (CHCl_3_), triethylamine (Et_3_N), and sulfuric acid (H_2_SO_4_) were purchased from Carlo Erba Reactifs SDS (Val de Reuil, France). Hexadeuterated dimethylsulfoxide (DMSO-*d*_6_) was purchased from Eurisotop (Saclay, France). Deionized water was used to prepare aqueous solutions.

### 3.2. NMR

NMR analyses were performed in DMSO-*d*_6_ at 298 K on an Avance AVIII-600 spectrometer (Bruker, Karlsruhe, Germany) equipped with a cryoprobe optimized for ^1^H detection and fitted with cooled ^1^H, ^13^C and ^2^H coils and preamplifiers. TopSpin 3.2 (Bruker, Karlsruhe, Germany) was used for data acquisition using standard microprograms. Data processing relied on TopSpin 4.0. The central resonance of DMSO-*d*_6_ (septet) was set at δ_C_ 39.8 for ^13^C-NMR spectrum referencing. The central resonance of residual DMSO-*d*_5_ (quintet) was set at δ_H_ 2.5 for ^1^H-NMR spectrum referencing.

### 3.3. UPLC-HRMS

Ultra Performance Liquid Chromatography coupled to Mass Spectrometry (UPLC-MS) analyses were performed with an Acquity UPLC H-Class (Waters, Manchester, UK) system coupled to a Synapt G2-Si (Waters) equipped with an electrospray (ESI) ion source. Chromatographic separation was achieved on a Uptisphere Strategy C18-HQ column (150 × 2.1 mm, 2.2 µm; Interchim, Montluçon, France). A gradient elution mode was used with solvent A (ammonium acetate 1%, pH 6.6) and solvent B (CH_3_CN) at flow rate of 0.4 mL min^−1^. Starting from 10%B, the gradient was linearly increased to 20%B in 6 min, then to 25%B in other 6 min, after 0.2 min the percentage of B was increased to 100% keeping it constant for 1 min. Finally, the gradient returned in the initial conditions in 0.2 min, maintaining it constant for 2 min for equilibration. The injection volume was 1 μL, the column temperature was regulated at 30 °C. All samples were solubilized in methanol and analyzed at concentration of 200 ppm. MS data acquisition parameters were: capillary voltage 3 kV, desolvation temperature 450 °C, desolvation gas flow 950 L/h, source temperature 120 °C, cone voltage 40 V, cone gas flow 50 L/h and scanning range of *m/z* 50–2000.

### 3.4. CPC

Centrifugal Partition Chromatography (CPC) fractionations were carried out using a lab-scale FCPE300® column of 303 mL inner volume (Kromaton Technology, Angers, France). The column was composed of 7 circular partition disks, each engraved with 33 twin-cells of 1.0 mL. The liquid phases were pumped by a preparative 1800 V7115 pump (Knauer, Berlin, Germany). Fractions of 20 mL were collected by a Labocol Vario 4000 (Labomatic Instruments, Allschwil, Switzerland). MtBE, CH_3_CN and H_2_O were equilibrated according the proportion 5:2:3 (*v/v*) and the two phases were separated. The lower aqueous phase was used as stationary phase and acidified with H_2_SO_4_ 10 mM (retainer). The upper organic phase was alkalinized with Et_3_N 8 mM (displacer) and used as mobile phase. The column was filled with the stationary phase at 300 rpm column rotation speed and 50 mL/min and then the mobile phase was pumped at 1200 rpm and 20 mL/min for hydrodynamic column equilibration. 1 g of extract was solubilized in 10 mL of retainer phase (acidified aqueous phase) and 5 mL of neutral organic phase. After sample loading through a 6-port Rheodyne valve (UpChurch Scientific, Oak Harbor, WA, USA) equipped with a 20 mL sample loop, the mobile phase was pumped into the column in ascending mode at flow-rate of 20 mL/min and 1200 rpm. The fractions were collected from the basic organic mobile phase and pooled according to TLC offline analysis to give 13 fractions namely A1–A13. TLC analysis was achieved on Merck TLC Silica gel 60 F254 plates, using CHCl_3_/ MeOH (8.5/1.5) as eluent. All experiments were conducted at room temperature (20 ± 2 °C).

### 3.5. Plant Material

Fresh bulbs of *U. peruviana* (1090.3 g) were purchased at the horticultural nursery Quatro Estaciones (Cochabamba, Bolivia) in August 2019. Some bulbs were grown, and the plants were identified by Dr. Umberto Mossetti, a voucher specimen (BOLO0602041) was deposited in the Herbarium of University of Bologna. The bulbs were stored in a cold room at 5 °C until the use, then they were freeze-dried and crushed, resulting in 220 g of plant material.

## 4. Conclusions

The rapid identification of natural products, either pure or in mixtures, depends on the availability of databases that connect together molecular structures, taxonomic information, and spectroscopic data, which constitute the three pillars of dereplication. We propose to the scientific community two easily findable NMR-based tools, the PNMRNP database that derives from earlier works on MS^2^ spectra prediction, and KnapsackSearch, a database generator that provides focused libraries of compounds whose content is oriented by biological taxonomy. These tools were involved in the study of an iberoamerican plant, *Urceolina peruviana*, in a way that relies strongly on ^13^C-NMR spectroscopy but also on other 1D and 2D NMR techniques as well as on preparative fractionation methods particularly suitable for alkaloid purification and on liquid chromatography coupled to high-resolution MS. The identification of five known compounds by these means is reported. The fully unambiguous characterization of a compound within a mixture may be reached only after purification and an exhaustive analytical study. However, the rapid and context adapted structure analysis is feasible by means of an approach that relies on computer databases and that adequately contributes to the study of complex natural substances.

## Data Availability

NMR time-domain and spectral data files are temporarily available (1.6 Gbytes) from http://eos.univ-reims.fr/LSD/OpenData_Molecules_2021_Nuzillard.zip and permanently available from https://doi.org/10.5281/zenodo.4309769 so that all acquisition and processing parameters may be freely consulted. These archives also contain the database files or ways to access them and files that were intermediately created for the composition of this article.

## Supplementary Materials

The following are available online, Figures S1 to S5 and Tables S1 to S2: titles as indicated in Table of Contents.
