# Supplementary material for: The Three Pillars of Natural Product Dereplication. Alkaloids from the Bulbs of Urceolina peruviana (C. Presl) J.F. Macbr. as a Preliminary Test Case
Source: Molecules. 2021 Jan 26;26(3):637. doi: 10.3390/molecules26030637 (PMC7865595; doi:10.3390/molecules26030637)
Supplement: Supplementary file 1 [file molecules-26-00637-s001.pdf]

**SUPPLEMENTARY INFORMATION TO:**

The three pillars of natural product dereplication. Alkaloids from the bulbs of *Urceolina peruviana* (C. Presl) J.F. Macbr. as a preliminary test case.

Mariacaterina Lianza [1], Ritchy Leroy [2], Carine Machado Rodrigues [2], Nicolas Borie [2], Charlotte Sayagh [2], Simon Remy [2], Stefan Kuhn [3], Jean-Hugues Renault [2], Jean-Marc Nuzillard [2]

1. University of Bologna, Department of Pharmacy and Biotechnology, 40126 Bologna, Italy

2. Université de Reims Champagne Ardenne, CNRS, ICMR UMR 7312, 51097 Reims, France

3. De Montfort University, School of Computer Science and Informatics, Leicester LE1 9BH, UK

**Table of Contents**

**Figure S1.** Step-by-step processing by KnapsackSearch of a list of organism genera (*family*_genera.txt, *family* being replaced by an organism family name or an alias, like amaryll standing for Amaryllidaceae) into an SDF file (*family*_knapsack.sdf) with automatically generated 2D structure, basic molecular properties, taxonomic data, and nmrshiftdb2-predicted $^{13}$C NMR chemical shifts. All scripts are available from https://github.com/nuzillard/KnapsackSearch.

**Figure S2. a.** Screenshot, response of http://www.knapsackfamily.com/knapsack_core/top.php when searching for Organism "Galanthus". The list of data items was truncated. **b**. Screenshot, response of http://www.knapsackfamily.com/knapsack_core/top.php when searching for C_ID C00001570 (galanthamine), upper part of screen, with molecular data and lower part with botanical data (truncated). **c.** Screenshot, view of galanthamine by EdiSDF as produced by KnapsackSearch and present in database DB2. The list of chemical shifts was truncated.

**Figure S3.** Comparison of the $^{13}$C NMR spectra of extracts 1, 2, and 3, drawn in sub-figures *a*, *b*, and *c*, respectively.

**Figure S4.** Procedure for the semi-automatic supplementation of ACD/Labs databases with predicted chemical shifts. ACD C+H NMR Predictors and DB 2020 and 2019.

**Figure S5.** Substructures that define eight classes of Amaryllidaceae alkaloids. Sub3 is a substructure of Sub5 and Sub1, thus reducing to six the effective number of substructures.

**Table S1.** Lists of $^{13}$C NMR chemical shifts drawn from the spectra of fractions A4, A7, A9, and A11. The number of attached protons, or H multiplicity, is given by symbols between parenthesis: s, d, t, and q for quarternary, methine, methylene, and methyl carbons, respectively. H multiplicity was derived from the inspection of the multiplicity-edited HSQC spectra of fractions.

**Table S2.** Tentative identification of compounds in DB1, DB2, DB3, DB3' and DB4 that fit with the $^{13}$C NMR chemical shifts from fractions F4, F7, F9, and F11.

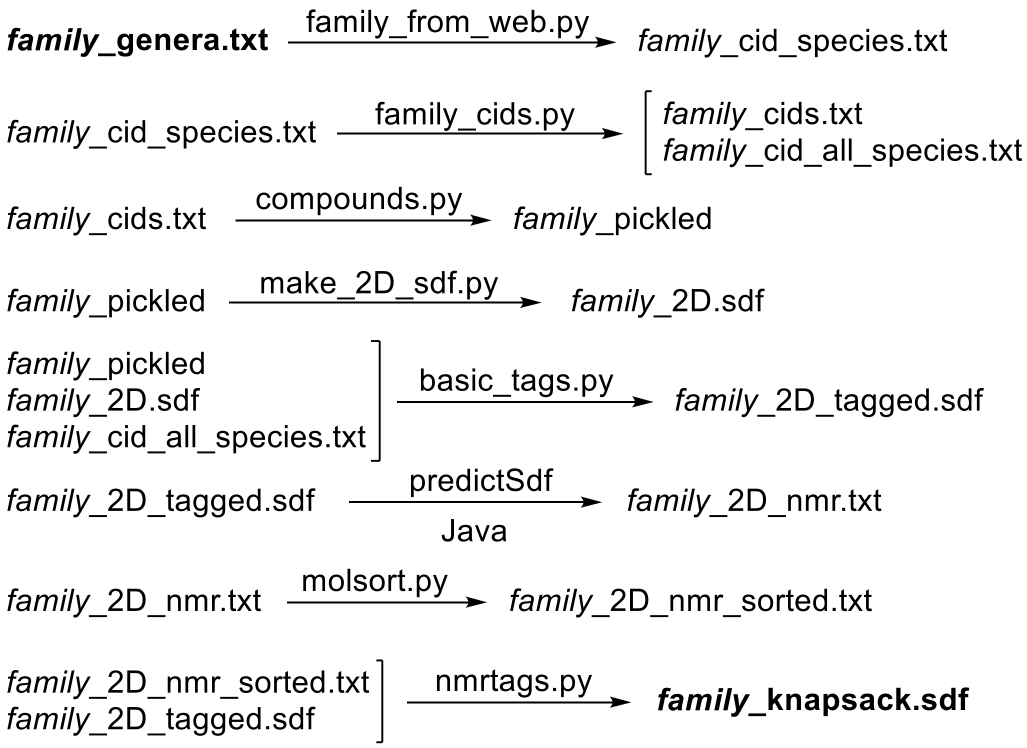


**Figure S1.** Step-by-step processing by KnapsackSearch of a list of organism genera (*family*_genera.txt, *family* being replaced by an organism family name or an alias, like amaryll standing for Amaryllidaceae) into an SDF file (*family*_knapsack.sdf) with automatically generated 2D structure, basic molecular properties, taxonomic data, and nmrshiftdb2-predicted $^{13}C$ NMR chemical shifts. All scripts are available from https://github.com/nuzillard/KnapsackSearch.

www.knapsackfamily.com/knapsack_core/result.php?sname=organism&word=galanthus

input type = organism , input word = galanthus

Number of matched data :76

| C_ID | CAS ID | Metabolite | Molecular formula | Mw | Organism |
|---|---|---|---|---|---|
| C00000575 | 484-20-8 | Bergaptan | C12H8O4 | 216.04225874 | Brachyclados megalanthus |
| C00001417 | 539-15-1 | Hordenine | C10H15NO | 165.11536411 | Galanthus plicatus spp.byzantinus |
| C00001417 | 539-15-1 | Hordenine | C10H15NO | 165.11536411 | Galanthus elewesii |
| C00001570 | 357-70-0 | Galanthamine | C17H21NO3 | 287.15214354 | Galanthus woronowii Losinsk. |
| C00001570 | 357-70-0 | Galanthamine | C17H21NO3 | 287.15214354 | Galanthus spp. |
| C00001570 | 357-70-0 | Galanthamine | C17H21NO3 | 287.15214354 | Galanthus elwesii Hook. |
| C00001570 | 357-70-0 | Galanthamine | C17H21NO3 | 287.15214354 | Galanthus elewesii |
| C00001570 | 357-70-0 | Galanthamine | C17H21NO3 | 287.15214354 | Galanthus caucasicus |
| C00001570 | 357-70-0 | Galanthamine | C17H21NO3 | 287.15214354 | Galanthus nivalis |
| C00001571 | 466-75-1 | Haemanthamine | C17H19NO4 | 301.1314081 | Galanthus elwesii |
| C00001572 | 477-17-8 | Hippeastrine | C17H17NO5 | 315.11067266 | Galanthus nivalis |
| C00001576 | 476-28-8 | Lycorine | C16H17NO4 | 287.11575804 | Galanthus woronowii Losinsk. |
| C00001576 | 476-28-8 | Lycorine | C16H17NO4 | 287.11575804 | Galanthus caucasicus |
| C00001576 | 476-28-8 | Lycorine | C16H17NO4 | 287.11575804 | Galanthus nivalis |
| C00001576 | 476-28-8 | Lycorine | C16H17NO4 | 287.11575804 | Galanthus elewesii |
| C00001578 | 510-77-0 | Narwedine | C17H19NO3 | 285.13649348 | Galanthus nivalis |
| C00001578 | 510-77-0 | Narwedine | C17H19NO3 | 285.13649348 | Galanthus elwesii |

**Figure S2a.** Screenshot, response of http://www.knapsackfamily.com/knapsack_core/top.php when searching for Organism "Galanthus". The list of data items was truncated.

www.knapsackfamily.com/knapsack_core/information.php?sname=C_ID&word=C0000157

input word = C00001570

| Metabolite Information | |
|---|---|
| Name | Galanthamine<br>(-)-Galanthamine<br>Jilkon<br>Lycoremin<br>Lycoremine |
| Formula | C17H21NO3 |
| Mw | 287.15214354 |
| CAS RN | 357-70-0 |
| C_ID | C00001570 , Twins (%) |
| InChIKey | ASUTZQLVASHGKV-KJOHYSOBNA-N |
| InChICode | InChI=1S/C17H21NO3/c1-18-8-7-17-6-5-12(19)9-14(17)21-16-13(20-2)4-3-11(10-18)15(16)17<br>/h3-6,12,14,19H,7-10H2,1-2H3/t12-,14-,17-/m0/s1 |
| SMILES | c1cc2c3c(c1OC)O[C@@H]1[C@@]3(CCN(C2)C)C=C[C@@H](C1)O |
| Start Substs in Alk. Biosynthesis (Prediction) | L-Tyr |

Structural formula

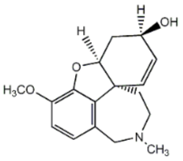


zoom in

Organism

| Kingdom | Family | Species | Reference |
|---|---|---|---|
| Plantae | Amaryllidaceae | Crinum spp. | Ref. |
| Plantae | Amaryllidaceae | Cyrtanthus elatus | Ref. |
| Plantae | Amaryllidaceae | Galanthus caucasicus | Ref. |
| Plantae | Amaryllidaceae | Galanthus elewesii | Ref. |
| Plantae | Amaryllidaceae | Galanthus elwesii Hook. | Ref. |
| Plantae | Amaryllidaceae | Galanthus nivalis | Ref. |
| Plantae | Amaryllidaceae | Galanthus spp. | Ref. |
| Plantae | Amaryllidaceae | Galanthus woronowii Losinsk. | Ref. |
| Plantae | Amaryllidaceae | Hippeastrum spp. | Ref. |
| Plantae | Amaryllidaceae | Hymenocallis spp. | Ref. |
| Plantae | Amaryllidaceae | Leucojum aestivum | Ref. |
| Plantae | Amaryllidaceae | Leucojum spp. | Ref. |
| Plantae | Amaryllidaceae | Leucojum vernum | Ref. |
| Plantae | Amaryllidaceae | Lycoris radiata Herb. | Ref. |
| Plantae | Amaryllidaceae | Lycoris sanguinea | Ref. |
| Plantae | Amaryllidaceae | Lycoris spp. | Ref. |
| Plantae | Amaryllidaceae | Narcissus confusus | Ref. |
| Plantae | Amaryllidaceae | Narcissus pseudonarcissus subsp.pseudonarcissus | Ref. |
| Plantae | Amaryllidaceae | Narcissus spp. | Ref. |
| Plantae | Amaryllidaceae | Pancratium spp. | Ref. |
| Plantae | Amaryllidaceae | Ungernia spp. | Ref. |

**Figure S2b**. Screenshot, response of http://www.knapsackfamily.com/knapsack_core/top.php when searching for C_ID C00001570 (galanthamine), upper part of screen, with molecular data and lower part with botanical data (truncated).

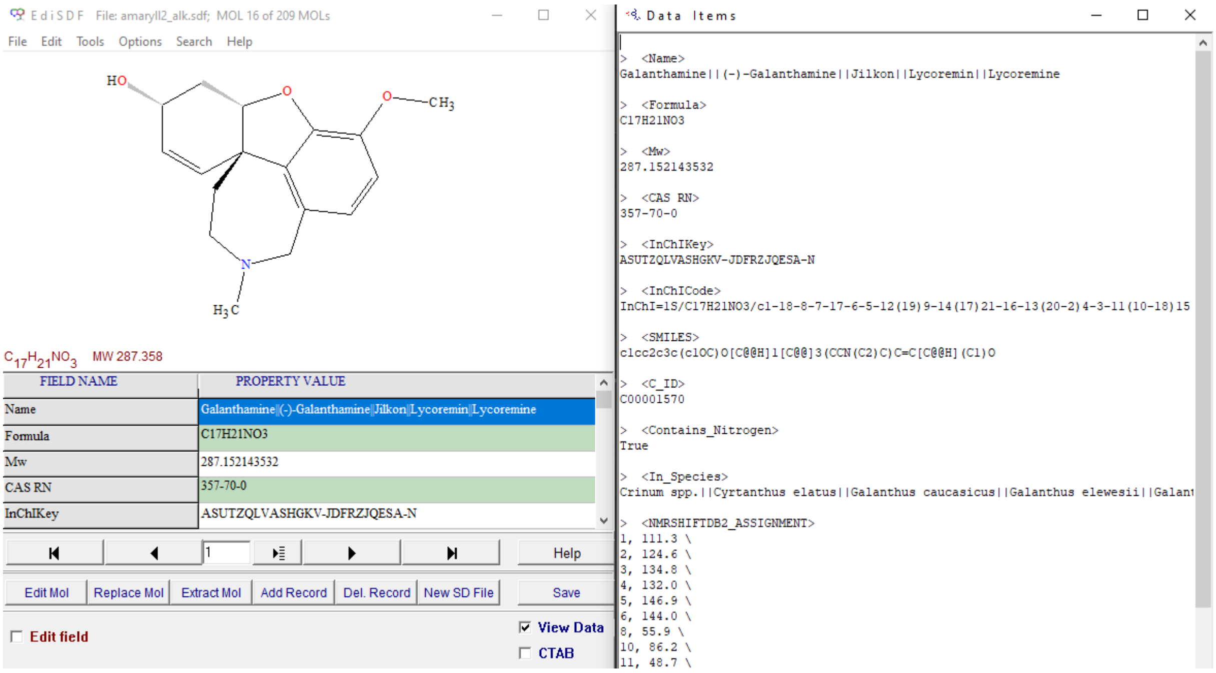


**Figure S2c.** Screenshot, view of galanthamine by EdiSDF as produced by KnapsackSearch and present in database DB2. The list of chemical shifts was truncated.

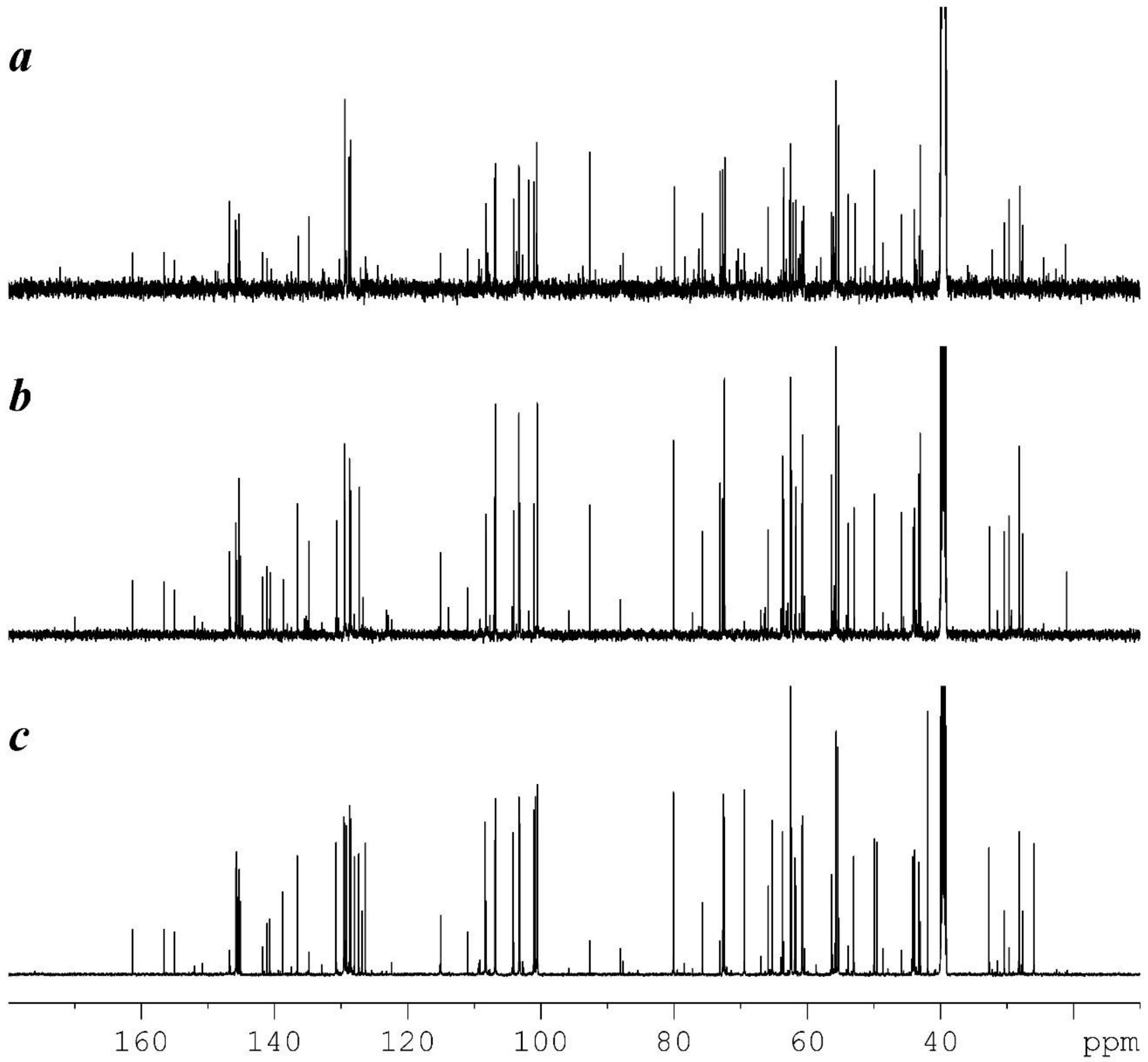


**Figure S3.** Comparison of the $^{13}C$ NMR spectra of extracts 1, 2, and 3, drawn in sub-figures *a*, *b*, and *c*, respectively.

1. Launch CNMR Predictor. ChemSketch window appears
2. Switch to Database window, bottom menu bar, item 7-Database. The Database interface appears
3. Create database. Database->New... from top menu bar. The "Create New Database" pop-up appears.
4. Indicate directory and name for the database location. Save. Leave passwords blank. OK.
5. Click "Database->Import" from top menu bar. The "Import" pop-up appears.
6. Indicate .sdf file directory and name. The "Import Options" pop-up appears
7. Check for "Search for Duplicated Chemical Structures" and select "Stop and Prompt". OK.
8. The "Import Information" pop-up apprears. OK. The first structure appears in the left-side panel
9. In the toolbar click on the "Copy Chemical Structure to Editor" button, on the left of the "Delete Record" button, the latter shows a red cross.
10. The ChemSketch window appears with the structure inside. Select All (Ctrl-A) and Copy (Ctrl-C) the structure.
11. Click on the "7-Database" button in the bottom menu bar. Back to the database panel.
12. Click on "ACD/Labs -> Spectrus". The Spectrus windows appears.
13. Paste the structure with Ctrl-V. The structure appears in the right-side panel.
14. Click on the pocket calculator icon at the very right of the tool bar of the right-side panel.
Possibly enlarge this panel to let the icon show up if hidden (This may happen).
15. Select HNMR under the calculator icon. The 1H-NMR spectrum and the parameter table show up in the center panel
16. Click "Database -> Update C+H NMR Database". The "Duplicated Structure" pop-up appears. Click "Update".
17. The database window pops-up with 1H NMR data tables in the center panel.
18. Click on "ACD/Labs -> Spectrus". The Spectrus windows appears.
19. Click on the pocket calculator icon at the very right of the tool bar of the right-side panel and select CNMR.
The 13C-NMR spectrum shows up in the center panel, under the 1H spectrum.
20. Click "Database -> Update C+H NMR Database". The "Duplicated Structure" pop-up appears. Click "Update".
21. The database window pops-up with 1H and 13C NMR data tables in the center panel.
22. Click on >> in the top tool bar to select the next molecule.
23. In the toolbar click on the "Copy Chemical Structure to Editor" button, on the left of the "Delete Record" button.
24. A pop-up appears to ask for deleting the existing structure in ChemSketch. Click the "Yes" button.
25. Select all and copy and go to the database window (7-Database, bottom menu bar). Select "ACD/Labs -> Spectrus"
26. Delete the two spectra, by clicking on the red cross that appears when the mouse hovers above the left-side orange spectrum bar.
Do not save the changes.
27. Copy the structure to the right panel of Spectrus with Ctrl-V and go back to step 14.
28. The process maybe stopped after step 21.
29. Click "Database -> Close" to close the database and "Database -> Exit" to exit the Database. The Spectrus database becomes visible
30. Click "File -> Exit" and then "No All". Spectrus and ChemSketch windows disappear.

**Figure S4.** Procedure for the semi-automatic supplementation of ACD/Labs databases with predicted chemical shifts. ACD C+H NMR Predictors and DB 2020 and 2019.

| Substructure | Type | SMARTS |
|---|---|---|
| 1 | Lycorine | Oc1cc2C~3~C~C~C~C~4~C~C~N(~C~3~4)~Cc2cc1O |
| 2 | Homolycorine | Oc1cc2C~3~C~4~N~C~C~C~4~C~C~C~3OC(~O)c2cc1O |
| 3 | Narciclassine | Oc1cc2C~3~C~C~C~C~C~3~N~Cc2cc1O |
| 4 | Galanthamine | Oc1ccc(c24)C~N~C~C~C2~3~C~C~C~C~C~3Oc14 |
| 5 | Crinine | Oc1cc2C~N(~C~C~4)~C~3~C~C~C~C~C~3~4c2cc1O |
| 6 | Tazettine | Oc1cc2COC~3~C~N~C~4~C~C~C~C~C~3~4c2cc1O |
| 7 | Norbelladine | c1cc(O)c(O)cc1C~N~C~Cc1ccc(O)cc1 |
| 8 | Montanine | Oc1cc2C~N(~C~3)~C~4~C~C~C~C~C~4~C~3c2cc1O |

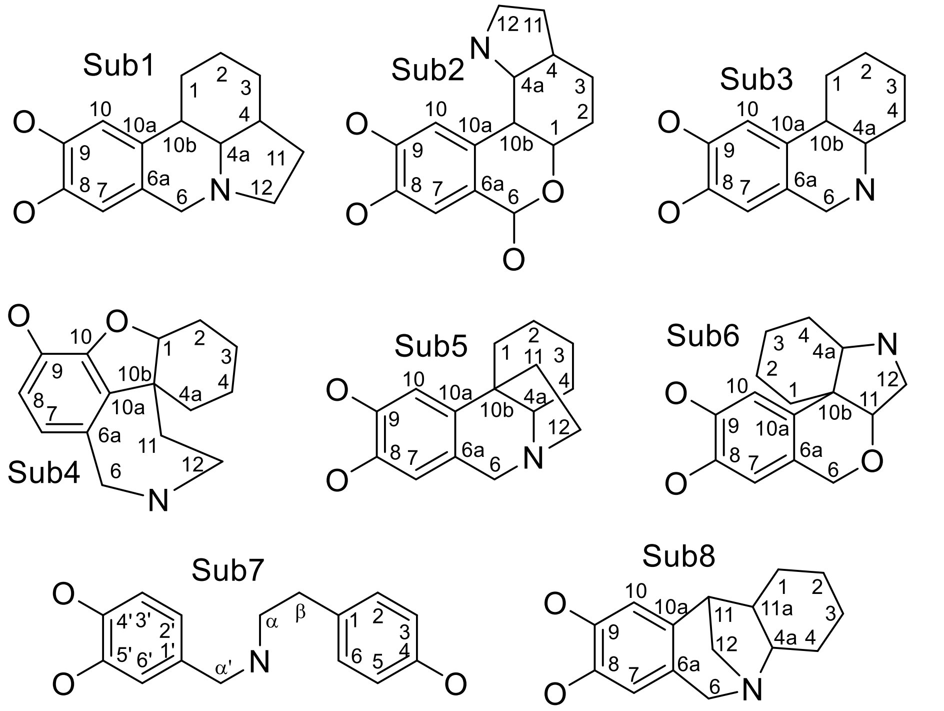


**Figure S5.** Substructures that define eight classes of Amaryllidaceae alkaloids. Sub3 is a substructure of Sub5 and Sub1, thus reducing to six the effective number of substructures.

| A4 | A7 | A9 | A11 |
|---|---|---|---|
| 145.7380(s) | 161.2988(s) | 145.7999(s) | 145.5792(s) |
| 145.6658(s) | 156.5513(s) | 145.2919(s) | 145.1041(s) |
| 129.5609(d) | 155.0277(s) | 136.5904(s) | 138.8210(s) |
| 129.1635(d) | 141.8143(s) | 129.5186(d) | 130.7921(d) |
| 127.9679(s) | 141.1331(s) | 128.7362(d) | 128.4712(d) |
| 126.3558(s) | 140.6848(s) | 127.3450(s) | 126.9639(s) |
| 108.3809(d) | 115.0101(d) | 106.8113(d) | 106.8801(d) |
| 104.1610(d) | 110.9643(s) | 103.2841(d) | 103.1479(d) |
| 101.0643(s) | 108.2002(d) | 100.5172(t) | 100.4552(t) |
| 100.8134(t) | 75.7086(d) | 80.0883(d) | 62.4607(d) |
| 72.6015(d) | 65.8267(d) | 72.4080(d) | 62.3959(d) |
| 69.4049(d) | 61.6679(q) | 63.7084(t) | 61.9272(t) |
| 65.2038(t) | 60.7180(q) | 62.4698(d) | 53.0113(t) |
| 60.7529(t) | 56.3367(q) | 60.7084(t) | 44.1980(t) |
| 55.3574(q) | 55.7209(t) | 55.6452(q) | 43.8661(s) |
| 49.4757(s) | 43.9295(d) | 49.8956(s) | 32.7112(t) |
| 41.8599(q) | 43.2234(q) | 28.1847(t) | |
| 25.9311(t) | 30.3999(t) | | |
| | 27.5896(t) | | |

**Table S1.** Lists of $^{13}C$ NMR chemical shifts drawn from the spectra of fractions A4, A7, A9, and A11. The number of attached protons, or H multiplicity, is given by symbols between parenthesis: s, d, t, and q for quarternary, methine, methylene, and methyl carbons, respectively. H multiplicity was derived from the inspection of the multiplicity-edited HSQC spectra of fractions.

| Fraction | Database | Minimum number of matching C atoms | Tolerance (ppm) | Number of solutions | Solutions | Filter 1 | Number of solutions | Solutions | Filter 2 | Number of solutions | Solutions |
|---|---|---|---|---|---|---|---|---|---|---|---|
| A4 | DB1 | 18 | 2 | 2 | Taezttine, Criwelline | | | | | | |
| | DB2 | | | 0 | | | | | | | |
| | DB3 | | | 22 | ==> | 18 C, 1-2 N | 7 | isomers of tazettine | | | |
| | DB3' | | | 7 | isomers of tazettine | | | | | | |
| | DB4 | | | 1 | Tazettine (flat) | | | | | | |
| A7 | DB1 | 19 | 2 | 1 | Albomaculine | | | | | | |
| | DB2 | | 2 | | | | | | | | |
| | | | 3 | 0 | | | | | | | |
| | | | 4 | | | | | | | | |
| | | 18 | 4 | 1 | Secoplicamine (C26) | 19 C, 1-2 N | 0 | | | | |
| | | 17 | | 5 | ==> | | 1 | Albomaculine | | | |
| | DB3 | 17 | 4 | 2750 | ==> | 19 C, 1-2 N | 39 | ==> | $C_{19}H_{23}NO_5$ | 2 | isomers of albomaculine |
| | | | | | | | | | $C_{19}H_{25}NO_5$ | 1 | 1-methylexcentricine |
| | DB3' | | 4 | 10 | ==> | 19 C, 1-2 N | 2 | isomers of albomaculine | | | |
| | DB4 | | | 28 | | | 1 | Albomaculine (flat) | | | |
| A9 | DB1 | 17 | 2 | 0 | | | | | | | |
| | | | 3 | 1 | Varadine (C18) | 17 C, 1-2 N | 0 | | | | |
| | | | 4 | 10 | ==> | | 5 | ==> | C17H19NO4 | 2 | isomers of haemanthamine |
| | DB2 | | 2 | 0 | | | | | | | |
| | | | 3 | 2 | isomers of haemanthamine | | | | | | |
| | DB3 | | 3 | 229 | ==> | 17 C, 1-2 N | 9 | isomers of haemanthamine | | | |
| | DB3' | | 3 | 9 | isomers of haemanthamine | | | | | | |
| | DB4 | | 3 | 18 | ==> | 17C, 1-2 N | 2 | haemanthamine, haemanthidine | C17H19NO4 | 1 | haemanthamine (flat) |
| A11 | DB1 | 16 | 2 | 1 | epi-vittatine | | | | | | |
| | | | 3 | 3 | ==> | 16 C, 1-2 N | 2 | epi-vittatine, caranine | | | |
| | DB2 | | 2 | 1 | caranine | | | | | | |
| | | | 3 | 13 | ==> | 16 C, 1-2 N | 4 | 3 isomers of crinine, caranine | | | |
| | DB3 | | 2 | 27 | ==> | 16 C, 1-2 N | 0 | | | | |
| | | | 3 | 219 | | | 6 | isomers of crinine | | | |
| | DB3' | | 2 | 0 | | | | | | | |
| | | | 3 | 42 | ==> | 16 C, 1-2 N | 6 | isomers of crinine | | | |
| | DB4 | | 2 | 0 | | | | | | | |
| | | | 3 | 17 | ==> | 16C, 1-2 N | 2 | crinine, caranine (flat) | | | |

**Table S2.** Tentative identification of compounds in DB1, DB2, DB3, DB3' and DB4 that fit with the $^{13}C$ NMR chemical shifts from fractions F4, F7, F9, and F11.
